# Soybean *GmMYB133* Inhibits Hypocotyl Elongation and Confers Salt Tolerance in *Arabidopsis*

**DOI:** 10.3389/fpls.2021.764074

**Published:** 2021-12-23

**Authors:** Binghui Shan, Wei Wang, Jinfeng Cao, Siqi Xia, Ruihua Li, Shaomin Bian, Xuyan Li

**Affiliations:** ^1^College of Plant Science, Jilin University, Changchun, China; ^2^Hebei Key Laboratory of Crop Salt-Alkali Stress Tolerance Evaluation and Genetic Improvement, Cangzhou, China; ^3^Academy of Agricultural and Forestry Sciences, Cangzhou, China

**Keywords:** *GmMYB133*, *RVE* gene, salt tolerance, soybean, hypocotyl elongation, *PRR5*

## Abstract

*REVEILLE* (*RVE*) genes generally act as core circadian oscillators to regulate multiple developmental events and stress responses in plants. It is of importance to document their roles in crops for utilizing them to improve agronomic traits. Soybean is one of the most important crops worldwide. However, the knowledge regarding the functional roles of *RVE*s is extremely limited in soybean. In this study, the soybean gene *GmMYB133* was shown to be homologous to the *RVE8* clade genes of *Arabidopsis*. *GmMYB133* displayed a non-rhythmical but salt-inducible expression pattern. Like *AtRVE8*, overexpression of *GmMYB133* in *Arabidopsis* led to developmental defects such as short hypocotyl and late flowering. Seven light-responsive or auxin-associated genes including *AtPIF4* were transcriptionally depressed by *GmMYB133*, suggesting that *GmMYB133* might negatively regulate plant growth. Noticeably, the overexpression of *GmMYB133* in *Arabidopsis* promoted seed germination and plant growth under salt stress, and the contents of chlorophylls and malondialdehyde (MDA) were also enhanced and decreased, respectively. Consistently, the expressions of four positive regulators responsive to salt tolerance were remarkably elevated by *GmMYB133* overexpression, indicating that *GmMYB133* might confer salt stress tolerance. Further observation showed that *GmMYB133* overexpression perturbed the clock rhythm of *AtPRR5*, and yeast one-hybrid assay indicated that GmMYB133 could bind to the *AtPRR5* promoter. Moreover, the retrieved ChIP-Seq data showed that AtPRR5 could directly target five clients including *AtPIF4*. Thus, a regulatory module *GmMYB133-PRR5-PIF4* was proposed to regulate plant growth and salt stress tolerance. These findings laid a foundation to further address the functional roles of *GmMYB133* and its regulatory mechanisms in soybean.

## Introduction

CIRCADIAN CLOCK ASSOCIATED 1 (CCA1)-like proteins are a subgroup of MYB-related family, which generally contain the consensus sequence SHAQK(Y/F)F within a single MYB repeat (Du et al., 2013). To date, numerous CCA1-like MYB transcription factors have been identified in different plant species. The best-known roles of *CCA1*-like *MYB* genes are their participation in circadian rhythm regulation, therefore enabling plants to make proper responses in the day/night time or under adverse environmental conditions (Carre and Kim, 2002; Nguyen and Lee, 2016). Basically, the morning expressed *CCA1*-like *MYB*s, *CCA1* and its homolog *LATE ELONGATED HYPOCOTYL 1* (*LHY1*), serve as central oscillators to control circadian rhythms directly through repressing the afternoon expressed *PSEUDO-RESPONSE REGULATOR* (*PRR*) genes. Sequentially, the midday-expressed *CCA1*-like *MYB*s, *REVEILLE* (*RVE*) genes such as *RVE4*, *RVE6*, and *RVE8*, can activate the expressions of the oscillator genes including *PRR*s and the evening-expressed genes (Nohales, 2021). Recently, it has been proposed that the balance between the activating and repressing MYB-like factors is more important in the regulation of the circadian clock than the presence or absence of any specific factor (Carre and Kim, 2002).

A substantial number of studies indicated that *CCA1*-like genes might be involved in a wide array of developmental processes including hypocotyl elongation, leaf senescence, flowering, seed germination, hormone signaling pathway, biosynthesis of anthocyanin and isoflavonoid, diurnal carbon allocation and growth, histone modifications, and iron homeostasis. For example, *LHY1* and *CCA1* regulate photoperiodic flowering in *Arabidopsis* (Muroya et al., 2021); *RVE4/6/8* in *Arabidopsis* repress plant growth by controlling the cell size (Gray et al., 2017); *RVE1/2* are able to regulate seed germination and dormancy (Yang et al., 2020); a few *CCA1*-like genes such as *MYBD* and *RVE8/LCL5* are positively involved in the regulation of anthocyanin biosynthesis (Nguyen et al., 2015; Perez-Garcia et al., 2015); *MYBH* participates in the mediation of hypocotyl elongation and leaf senescence (Kwon et al., 2013; Huang et al., 2015). Moreover, increasing evidence revealed that *CCA1*-like genes perform important functions in stress responses. For instance, *CCA1* enables to control the homeostasis of reactive oxygen species and oxidative stress response in *Arabidopsis* (Lai et al., 2012); *MYBS3* in rice is required for cold tolerance (Su et al., 2010); overexpression of *SgRVE6* confers physiological responses to cold stress in tobacco (Chen S. et al., 2020). More importantly, some regulatory mechanisms mediated by *CCA1*-like genes have been revealed in different plant species. For example, *RVE4/8* are able to govern plant thermotolerance through regulating the expression of *ETHYLENE RESPONSIVE FACTOR53* (*ERF53*) and *ERF54* (Li et al., 2019a); *RVE4/8* can be transferred from the cytoplasm to the nucleus, therefore, directly activating *DREB1* expression to confer cold tolerance (Kidokoro et al., 2021); *CCA1/LHY1*-mediated outputs from circadian clock contribute to plant cold tolerance *via* affecting the *CBF* cold-response pathway (Dong et al., 2011). Evidently, *CCA1*-like *MYB*s play important roles in diverse biological processes. However, the understanding of their functional roles is limited to a few plant species such as *Arabidopsis* and rice. Therefore, more efforts are needed to understand the functional roles of *CCA1*-like genes in diverse plant species.

Noticeably, several reports revealed that different members of the *CCA1*-like subfamily might perform different, even opposing, regulatory functions. For example, three members of the *RVE8* clade genes (*RVE4*, *RVE6*, and *RVE8*) in *Arabidopsis* promote clock pace in a partially redundant manner, while the remaining members, *RVE3* and *RVE5*, play only minor roles in the regulation of clock function (Gray et al., 2017); two *CCA1*-like genes in *Arabidopsis*, *MYBS1* and *MYBS2*, show converse roles in regulating glucose and abscisic acid (ABA) signaling during seed germination and early seedling development (Chen et al., 2017); *CCA1* represses *TOC1* expression by promoting histone deacetylation, whereas *RVE8/LCL5* might facilitate *TOC1* expression *via* pronouncing histone acetylation at the *TOC1* promoter region (Farinas and Mas, 2011). More interestingly, recent studies indicated that *CCA1*-like counterparts in different plant species sometimes show distinct or opposite functional roles. For example, two *LHY1* genes (*GmLHY1a* and *GmLHY1b*) in soybean (*Glycine max*) enable to decrease drought tolerance through suppressing ABA responses (Wang et al., 2021). However, in *Arabidopsis*, *LHY1* not only inhibits the expression of the rate-limiting enzyme gene (*9-CIS-EPOXYCAROTENOID DIOXYGENASE*) of ABA biosynthesis but also activates ABA-responsive genes required for drought tolerance (Adams et al., 2018). Thus, it is of significance to document the roles of individual *CCA1*-like family members in different plant species.

Soybean is well-recognized for the rich source of seed protein and edible oil as well as health-promoting compounds. Previously, we identified 54 soybean *CCA1*-like genes by data mining against the soybean genome database (Bian et al., 2017a). However, only few soybean *CCA1*-like genes have been functionally studied such as *GmLHY*s, *GmLCL*s, *GmMYB138a*, *GmMYB176*, and *GmMYB177*, which are associated with the regulation of plant height and internode, isoflavonoid accumulation, ABA perception, and signaling pathway, or in response to stresses (Liao et al., 2008; Li et al., 2012; Bian et al., 2017a; Wang et al., 2021; Yuan et al., 2021). Recently, *GmMYB133* was reported to be involved in the regulation of isoflavonoid accumulation (Bian et al., 2018). In this study, additional functional roles of *GmMYB133* were addressed in *Arabidopsis*. Overexpression of *GmMYB133* in *Arabidopsis* led to decreased hypocotyl length of seedlings, short leaf petiole, and late flowering as well as enhancement of salt tolerance during seed germination and plant growth. Consistently, nine auxin-associated genes, two light-responsive genes, and four salt tolerance-related genes were transcriptionally altered by *GmMYB133* overexpression. Yeast one-hybrid assay indicated that GmMYB133 can bind to the promoter region of *AtPRR5*, and the rhythmic expression of *AtPRR5* was extremely disrupted by *GmMYB133* overexpression. Moreover, the retrieved ChIP-Seq data indicated that AtPRR5 can directly target its client genes such as *AtCCA1*, *AtLHY1*, *AtPIF4*, *AtBBX24*, and *AtIAA19*. These findings provided solid information to address the functional roles of *GmMYB133* and its regulatory mechanism in soybean, especially hypocotyl elongation and salt tolerance.

## Materials and Methods

### Sequence Analysis and Alignment

The protein sequences of GmMYB133 and other CCA1-like transcription factors in soybean and *Arabidopsis* were downloaded from Phytozome^1^ and TAIR^2^. Multiple sequence alignment was performed by MEGA X and DNAMAN. The protein homology analysis was calculated using the online tool MUSCLE of EMBL-EBI^3^, and the heat map of the identity was generated using the TBtools software (Chen C. et al., 2020). The phylogenetic tree of GmMYB133 and other CCA1-like transcription factors was constructed by the maximum likelihood method with 1,000 bootstrap replications using MEGA X (Kumar et al., 2018). The number on the line indicates the branch length.

### Plant Materials

The *Arabidopsis* Col-0 and soybean (cultivar “Jilin 32”) were used in this study. Generally, *Arabidopsis* plants were grown in growth chambers under long days (16 h light/8 h dark) at 20°C with 70–80% relative humidity. To generate transgenic lines overexpressing *GmMYB133*, the full-length cDNA of *GmMYB133* was amplified and cloned into the Gateway vector pEarleyGate101 through recombination. Transgenic *Arabidopsis* was generated using the floral dip method (Tsuda et al., 2012). Positive transgenic lines were screened on murashige and skoog (MS) with 10 μg/ml of glufosinate, followed by real-time PCR (RT-PCR) for confirmation with gene-specific primers. Primer information is listed in Supplementary Table 1.

To generate β-glucuronidase (*GUS*) reporter lines, the 1,605 bp fragment of ATG upstream of *GmMYB133* was cloned and constructed into the Gateway vector pMDC162 to obtain *pGmMYB133:GUS*, and the floral dip method (Tsuda et al., 2012) was used to generate transgenic *Arabidopsis*. Positive transgenic lines were screened on MS with 50 μg/ml of hygromycin, followed by RT-PCR for confirmation. Primer information is listed in Supplementary Table 1.

### Histochemical and Fluorometric β-Glucuronidase Assays

To conduct histochemical GUS assay, the transgenic samples with *pGmMYB133:GUS* (7-day-old seedling, root, rosette leaf, cauline leaf, inflorescences, pod wall, and seeds) were collected and immersed in the GUS staining solution as previously described (Bian et al., 2017b). After vacuuming for 15 min, the samples were incubated in staining solution at 37°C overnight and then destained with 75% ethanol. To investigate the expression of *GmMYB133* under drought and salt, 7-day-old transgenic *Arabidopsis* seedlings with *pGmMYB133:GUS* were separately transferred to MS solid medium with 150 mM NaCl or 300 mM mannitol, and samples were collected for histochemical GUS staining and GUS activity assay at 3 days after stress treatment, respectively.

Fluorometric GUS assay was performed according to the method described by Jefferson et al. (1987). In brief, *Arabidopsis* seedlings were homogenized in 1 ml extraction buffer and centrifuged at 12,000 rpm for 10 min at 4°C. Notably, 100 μl supernatant was used to perform the assay of GUS activity in 400 μl reaction buffer with 1 mM 4-methylumbelliferyl-β-D-glucuronide. Fluorescence was measured using a fluorescence spectrophotometer at an excitation/emission wavelength of 365/455 nm. The protein concentration in the extracts was determined according to the method of bicinchoninic acid (BCA) assay (Smith et al., 1985). GUS activity was counted as picomoles of 4-methylumbelliferone per minute per milligram of protein.

### Treatment of Soybean and *Arabidopsis* for the Analysis of Circadian Rhythm

For the analysis of circadian rhythm, vernalized *Arabidopsis* seeds of untransformed (UT) control and T3 transgenic plants overexpressing *GmMYB133* as well as soybean seeds were sown and grown under 12 h light/12 h dark at 22°C for 10 days and then transferred into the condition of constant light. Samples of soybean and *Arabidopsis* were collected every 4 h from 0 to 72 h, respectively, and then frozen in liquid nitrogen and stored at −80°C for RNA extraction.

### Abiotic Stress Treatments of Soybean Seedling

Ten-day-old soybean seedlings were subjected to the treatments of cold, drought, and salt stresses as previously described (Li et al., 2015): For cold stress, the ground-above tissues were harvested at 0, 6, 12, 24, 48, and 72 h after seedlings were transferred to 4°C; for drought stress, the ground-above tissues were collected at 0, 2, 4, 6, 8, and 10 days of water stress; for salinity stress, the ground-above tissues were harvested at 0, 6, 12, 24, 48, and 72 h after 200 mM NaCl solution was applied to seedlings. Then, the collected samples were frozen in liquid nitrogen and stored at −80°C for RNA extraction. Each treatment had at least six pots of seedlings, and three biological replicates were performed for each treatment.

### Hypocotyl Length Assay

*Arabidopsis* seeds from *GmMYB133*-overexpressing transgenic lines and UT control were sterilized and planted on MS solid medium, kept at 4°C for 2 days, and grown in a growth chamber under 16 h light/8 h dark at 22°C. At least 30 9-day-old seedlings were used for hypocotyl length assay, and three biological replicates were performed. Statistical significance of the data was analyzed using independent-samples *t*-test. Error bars indicate SE and *p*-value < 0.01 (**).

### Analysis of Salt Tolerance Using Transgenic *Arabidopsis*

Germination assay of *Arabidopsis* seeds was conducted as previously described (Li et al., 2018). In brief, *Arabidopsis* seeds from *GmMYB133*-overexpressing transgenic lines and UT control were sterilized and sown on MS solid medium with or without 150 mM NaCl, kept at 4°C for 2 days, and then cultured under a photoperiod of 16/8 h (light/dark) at 22°C. The germination rates were monitored for 10 days. Data were analyzed using Graphpad Prism 8 software^4^.

To investigate the response of adult plants to salt stress, 3-week-old transgenic *Arabidopsis* plants and UT control were supplied with or without 200 mM NaCl, respectively. After salt stress was applied to *Arabidopsis* plants for 9 days, samples were collected for the extraction and determination of MDA and total chlorophylls. Additionally, 7-day-old transgenic *Arabidopsis* plants and UT control were subjected to cold stress (−9°C) for 1 h according to the procedure described by Jiang et al. (2020), and phenotypic difference and MDA content were investigated.

Extraction and determination of MDA were conducted according to the method described by Wu et al. (2021). In brief, 1 g of leaves from *GmMYB133*-overexpressing transgenic lines and UT control were homogenized in 10% trichloroacetic acid (TCA) and centrifuged for supernatant, which subsequently was mixed with 0.6% thiobarbituric acid in 10% TCA. The mixture was boiled for 15 min and placed on ice immediately, followed by a centrifuge at 10,000 *g* for 20 min. The absorbance of supernatants was spectrophotometrically measured at 450, 532, and 600 nm, and the following formula was used to count the MDA content of each supernatant: [6.459 × (A532 − A600) − 0.569 × A450]/fresh weight. Chlorophylls were extracted from leaves using acetone. Total chlorophyll content was spectrophotometrically measured as previously described (Li X. Y. et al., 2020).

Three biological replicates were performed with three-technique replicates for the extraction and determination of MDA and chlorophylls. Statistical significance of the data was analyzed using independent-samples *t*-test. Error bars indicate SE and *p*-value < 0.01 (**).

### RNA Extraction and Quantitative Real-Time PCR Analysis

Total RNAs of *Arabidopsis* and soybean were extracted using OminiPlant RNA Kit (CWBIO, China) and RNAprep Pure Plant Plus Kit (TIANGEN, China), respectively. The first-strand cDNA was generated using StarScript II First-strand cDNA Synthesis Mix With gDNA Remover Kit (GenStar, China). Quantitative real-time PCR (qRT-PCR) was performed using the Bio-Rad CFX Connect Real-Time PCR Detection System with the reagent of 2 × RealStar Green Fast Mixture (GenStar, China). *AtACTIN8* and *GmUBIQUITIN-3* (*GmSUBI3*) were used as the internal reference for *Arabidopsis* and soybean, respectively. The data were analyzed using Bio-Rad CFX Manager. Three biological replicates with three techniques were conducted for each sample. Primer information is listed in Supplementary Table 1. Statistical significance of the data was analyzed using independent-samples *t*-test. Error bars indicate SE and *p*-value < 0.05 (*) or < 0.01 (**).

### Yeast One-Hybrid Assay

The evening element was predicted using the online program PlantPAN 3.0 with TFmatrixID_0029 and TFmatrixID_0030.^5^

To perform Yeast One-Hybrid (Y1H) assay, the full-length cDNAs of two genes (*GmMYB133* and *AtPRR5*) and the promoter fragments of eleven genes (*AtPRR5*, *AtPIF4*, *AtBBX24*, *AtIAA19*, *AtIAA29*, *AtSAUR21*, *AtSAUR26*, *AtCAX3, AtEARLI1, AtMPK3*, and *AtAZI1*) were amplified using gene-specific primers (Supplementary Table 1). The full-length cDNAs of *GmMYB133* and *AtPRR5* were constructed into the vector of pB42AD as baits (pB42AD-GmMYB133 and pB42AD-AtPRR5), while promoter fragments of the above eleven genes were cloned into the vector pLacZi as preys [pLacZi-*pAtPRR5-1* and pLacZi-*pAtPRR5-2*, pLacZi-*pAtPIF4(EE)*, pLacZi-*pAtPIF4(G-box)*, pLacZi-*pAtBBX24*, pLacZi-*pAtIAA19*, pLacZi-*pAtIAA29*, pLacZi-*pAtSAUR21*, pLacZi-*pAtSAUR26*, pLacZi-*pAtCAX3*, pLacZi-*pAtEARLI1*, pLacZi-*pAtMPK3*, and pLacZi-*pAtAZI1*]. Different plasmid combinations were separately co-transfected into yeast competent cell EGY48, including pB42AD-GmMYB133/pLacZi-*pAtPRR5-1*, pB42AD-GmMYB133/pLacZi-*pAtPRR5-2*, pB42AD-GmMYB133/pLacZi-*pAtPIF4(EE)*, pB42AD-GmMYB133/pLacZi-*pAtPIF4(G-box)*, pB42AD-GmMYB133/pLacZi-*pAtBBX24*, pB42AD-GmMYB133/pLacZi-*pAtIAA19*, pB42AD-GmMYB133/pLacZi-*pAtIAA29*, pB42AD-GmMYB133/pLacZi-*pAtSAUR21*, pB42AD-GmMYB133/pLacZi-*pAtSAUR26*, pB42AD-GmMYB133/pLacZi-*pAtCAX3*, pB42AD-GmMYB133/pLacZi-*pAtEARLI1*, pB42AD-GmMYB133/pLacZi-*pAtMPK3*, pB42AD-GmMYB133/pLacZi-*pAtAZI1*, sixteen negative controls (pB42AD/pLacZi, pB42AD-GmMYB133/pLacZi, pB42AD-AtPRR5/pLacZi, pB42AD/pLacZi-*pAtPRR5-1*, pB42AD/pLacZi-*pAtPRR5-2*, pB42AD/pLacZi-*pAtPIF4-EE*, pB42AD/pLacZi-*pAtPIF4-G-Box*, pB42AD/pLacZi-*pAtBBX24*, pB42AD/pLacZi-*pAtIAA19*, pB42AD/pLacZi-*pAtIAA29*, pB42AD/pLacZi-*pAtSAUR21*, pB42AD/pLacZi-*pAtSAUR26*, pB42AD/pLacZi-*pAtCAX3*, pB42AD/pLacZi-*pAtEARLI1*, pB42AD/pLacZi-*pAtMPK3*, and pB42AD/pLacZi-*pAtAZI1*), and one positive control (pB42AD-AtRVE8/placZi-*pAtPRR5*).

### Retrievement and Visualization of AtPRR5 Targets

The ChIP-Seq data of AtPRR5 (Nakamichi et al., 2012) were searched and obtained against *Arabidopsis thaliana* using AtPRR5 as an assayed protein with the Encyclopedia of Plant Genome (ENPG) database.^6^ Enrichment peaks for the binding of AtPRR5 to the genomic regions of *AtLHY1*, *AtCCA1*, *AtPIF4*, *AtIAA19*, and *AtBBX24* were retrieved from the ChIP-Seq data of AtPRR5 and visualized through the program ENPG using the default parameters.

## Results

### Sequence Analysis of GmMYB133 in Soybean

**FIGURE 1:**
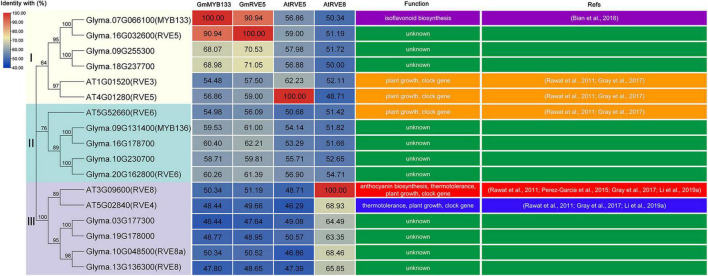
Sequence analysis of GmMYB133 and its homologous proteins in soybean and *Arabidopsis*. Phylogenetic relationship (left panel) and protein identities (middle panel) of GmMYB133 with its homologs as well as their functional roles (right panel), which were experimentally validated in previous studies. These proteins were clustered into three groups (I, II, and III); the numbers in the middle panel represent the percentage identity between proteins; the color scale and the numbers indicate the identities of GmMYB133, GmRVE5, AtRVE5, and AtRVE8 with their homologs; and low identity is indicated by blue color and high identity is indicated by red color.

Previously, GmMYB133 was revealed as a CCA1-like player in the regulation of isoflavonoid accumulation (Bian et al., 2018). To provide additional cues of its functional roles, we searched its homologs in soybean and *Arabidopsis* against the Phytozome and TAIR databases using the amino-acid sequence of GmMYB133 as a query, and sixteen proteins were obtained accordingly, e.g., GmRVE5/6/8/8a and AtRVE3/4/5/6/8. Multiple sequence alignment indicated that GmMYB133 and its homologs harbor a conserved MYB domain and the consensus sequence SHAQ(Y/F)F (Supplementary Figure 1). Phylogenetic analysis showed that all the MYB proteins were clustered into three groups, and GmMYB133 was distributed at the same clade with GmRVE5 (Supplementary Figure 1). In soybean, the protein identities between GmMYB133 and its homologs ranged from 46.44 to 90.94%, and GmMYB133 shared the maximum protein identity with GmRVE5 (Figure 1 and Supplementary Figure 1), which has not been functionally characterized. In *Arabidopsis*, GmMYB133 was closely related to the RVE8 clade proteins (AtRVE3, AtRVE4, AtRVE5, AtRVE6, and AtRVE8) known as the players to control hypocotyl elongation, flowering, leaf petiole growth, or respond to abiotic stresses (Perez-Garcia et al., 2015; Gray et al., 2017; Li et al., 2019a). The identities between GmMYB133 and the AtRVE8 clade genes were up to 48.44%–56.86% at the protein level with AtRVE5 showing maximum identity (Figure 1).

### Spatial and Diurnal Expression Patterns of *GmMYB133* and Its Response to Abiotic Stresses

**FIGURE 2:**
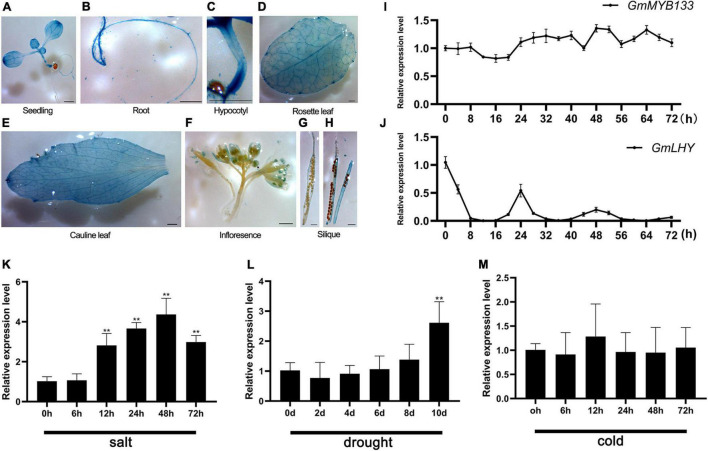
Spatial and diurnal expression patterns of *GmMYB133* and its response to abiotic stresses. **(A–H)** Histochemical analysis of *pGmMYB133:GUS* activity in *Arabidopsis*. Transgenic plants were used for analysis including 7-day-old seedling, root, rosette leaf, cauline leaf, inflorescences, pod wall, and seeds. Scale bars indicate 1 mm. **(I–J)** Diurnal expression patterns of *GmMYB133* and *GmLHY* (*Glyma.16G017400*) in soybean seedlings. Ten-day-old soybean seedlings were transferred into the condition of constant light, and the time course was set every 4 h from 0 h until 72 h. **(K–M)** Expression analysis of *GmMYB133* in response to abiotic stresses. Ten-day-old soybean seedlings were exposed to salinity **(K)** and cold **(M)** stresses for 0, 6, 12, 24, 48, and 72 h and drought stress for 0, 2, 4, 6, 8, and 10 d **(L)**. Error bars indicate SEs of three biological and three technical replicates. Values were normalized against the gene *GmSUBI3*. Significant differences are denoted by asterisks: **P < 0.01.

Previously, the expression pattern of *GmMYB133* was investigated in root, stem, leaves, nodules, flowers, and differently developing embryos (Bian et al., 2018). To explore the spatial expression pattern of *GmMYB133*, its promoter region was cloned and fused with the *GUS* reporter gene. The transgenic *Arabidopsis* lines were generated for histochemical staining. As shown in Figures 2A–H, GUS signal was clearly detected in seedling, root, hypocotyl, rosette leaf, cauline leaf, stigma, anther, and pod wall. Further observation indicated that the GUS signal mainly appeared in the vasculature of cotyledons, leaves, and roots. Since *GmMYB133* is homologous to the *AtRVE8* clade genes, the rhythmic oscillation of *GmMYB133* expression was examined under the constant light condition. The gene *GmLHY* (*Glyma.16G017400*) was set as a positive control. Consequently, although *GmMYB133* showed oscillating expression to some extent, 24 h rhythmicity was not observed for its expression (Figure 2I). As expected, a typical rhythmic expression was observed for *GmLHY* (Figure 2J).

Several studies showed that *CCA1*-like *MYB*s play important roles in response to diverse stresses (Lai et al., 2012; Li et al., 2019a; Chen S. et al., 2020; Yuan et al., 2021). To investigate if *GmMYB133* is involved in response to abiotic stresses, its expression pattern was determined under cold, drought, and salt stresses. When soybean seedlings were subjected to 200 mM NaCl stress, the transcript accumulation of *GmMYB133* was gradually increased as stress prolongs and reached its maximum level at 48 h after salt stress with 5.69-fold changes as compared with control (Figure 2K). When soybean seedlings were exposed to drought stress, the expression of *GmMYB133* remained a stable level until 10 days after stress, and its transcript accumulation was pronounced by 2.25-fold as compared with control (Figure 2L). However, *GmMYB133* showed no significant change when cold stress was applied to soybean seedlings (Figure 2M). Furthermore, the above GUS reporter *Arabidopsis* was used to investigate the expression of *GmMYB133* under drought and salt stress. As compared with the normal condition, a stronger GUS signal was observed in young leaves of seedlings under salt stress. No obvious difference was shown between normal condition and drought stress (Supplementary Figure 2A). Meanwhile, GUS activity was measured under drought and salt stress using the whole seedlings. As shown in Supplementary Figure 2B, no significant difference was observed for GUS activity between normal condition and salt stress as well as drought stress. Subsequently, the young leaves of the above seedlings were collected to investigate the expression of the *GUS* gene using the qRT-PCR approach. As compared with the normal condition, the expression of the *GUS* gene was significantly enhanced under both salt and drought stress. Especially under salt stress, a 149.5-fold increase was observed (Supplementary Figure 2C).

### *GmMYB133* Negatively Affects Hypocotyl Elongation in *Arabidopsis*

**FIGURE 3:**
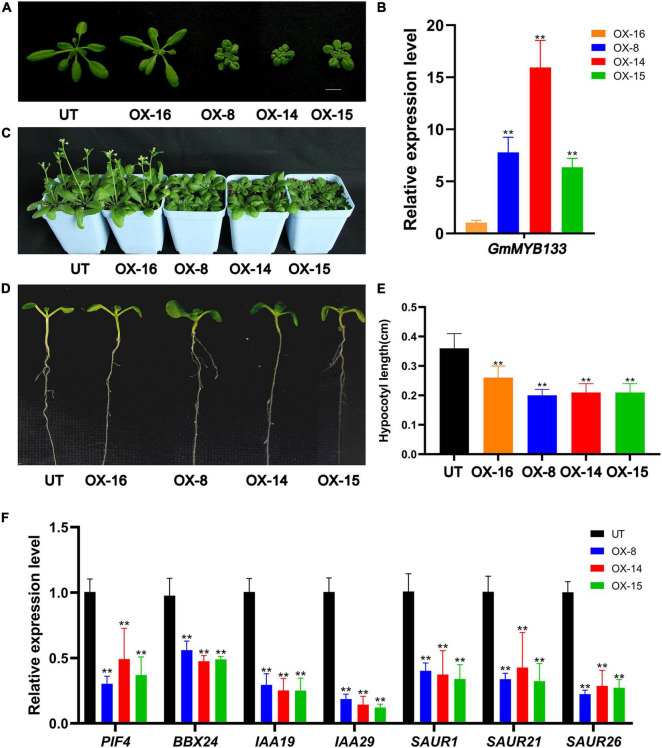
*GmMYB133* negatively affects hypocotyl elongation in *Arabidopsis*. **(A)** Photograph of untransformed (UT) plant and four transgenic *Arabidopsis* lines overexpressing *GmMYB133* (OX-16, OX-8, OX-14, and OX-15). **(B)** The expression of *GmMYB133* in UT and four transgenic *Arabidopsis* lines. The expression level in the transgenic line OX-16 was set as 1. **(C)** Photograph of 36-day-old plants of UT and four transgenic *Arabidopsis* lines. **(D,E)** Photograph of hypocotyl elongation **(D)** and hypocotyl length assay **(E)** of UT and four transgenic lines. Nine-day-old seedlings were used for the hypocotyl length assay. **(F)** The expression patterns of light-responsive genes and auxin-associated genes in UT and three transgenic *Arabidopsis* lines (e.g., *AtPIF4*, *AtBBX24*, *AtIAA19*, *AtIAA29*, *AtSAUR1*, *AtSAUR21*, and *AtSAUR26*). Total RNAs for **(B,F)** were exacted from 14-day-old transgenic seedlings and UT. Values were normalized against the gene *AtACTIN8*, and the expression level in UT was set as 1. Error bars in **(B,E,F)** indicate SE of three biological and technical replicates, and significant differences are denoted by asterisks: **p < 0.01.

To investigate its functional roles, *GmMYB133*-overexpressing *Arabidopsis* lines were generated. Four transgenic lines (named as OX-16, OX-8, OX-14, and OX-15) were chosen as representatives for further study. As shown in Figure 3A, transgenic plants showed a compact structure with short-petiole leaf, especially OX-8, OX-14, OX-15, and qRT-PCR analysis indicated that *GmMYB133* was, in fact, overexpressed in the four transgenic lines (Figure 3B). Consistent with the structural compactness of transgenic lines, a relatively low level of *GmMYB133* transcripts was observed in the transgenic line OX16 and high level in OX-8, OX-14, and OX-15 (Figures 3A,B). Further observation indicated that the transgenic lines OX-8, OX-14, and OX-15 showed a clear delay of flowering under the long-day condition (Figure 3C). Noticeably, the hypocotyl lengths of OX-16, OX-8, OX-14, and OX-15 seedlings were decreased to 72.2%, 55.6%, 58.3%, 58.3% of UT control (Figures 3D,E), respectively, suggesting that overexpression of *GmMYB133* might inhibit hypocotyl elongation in *Arabidopsis*.

Numerous studies indicated that endogenous auxin and exogenous light signal antagonistically regulate hypocotyl growth (Xi et al., 2021). To provide some clues about how *GmMYB133* can be involved in the regulation of hypocotyl elongation, seven genes were chosen to conduct expression analysis using the qRT-PCR approach, including two light-responsive genes (*AtPIF4* and *AtBBX24*) and five auxin-associated genes such as *AtIAA19*, *AtIAA29*, *AtSAUR1*, *AtSAUR21*, and *AtSAUR26*. Molecular and/or genetic study has shown that *AtPIF4*, *AtBBX24*, *AtIAA19*, *AtIAA29*, and *AtSAUR21* are positive regulators for hypocotyl elongation (Jiang et al., 2012; Spartz et al., 2012; Zhou et al., 2013; Shimizu et al., 2016; Li et al., 2021). As shown in Figure 3F, all the seven genes (*AtPIF4*, *AtBBX24*, *AtIAA19*, *AtIAA29*, *AtSAUR1*, *AtSAUR21*, and *AtSAUR26*) were transcriptionally suppressed by *GmMYB133* overexpression. For example, the expressions of *AtPIF4*, *AtIAA19*, and *AtIAA29* in three *GmMYB133*-overexpressing lines were decreased to 30.1%–49.1%, 25.0%–29.5%, and 12.1%–18.6% of UT control, respectively (Figure 3F). These observations suggested that *GmMYB133* might act as a negative regulator in the regulation of hypocotyl elongation through governing the expressions of light-responsive and auxin-associated genes.

### *GmMYB133* Confers Plant Tolerance to Salt Stress in *Arabidopsis*

**FIGURE 4:**
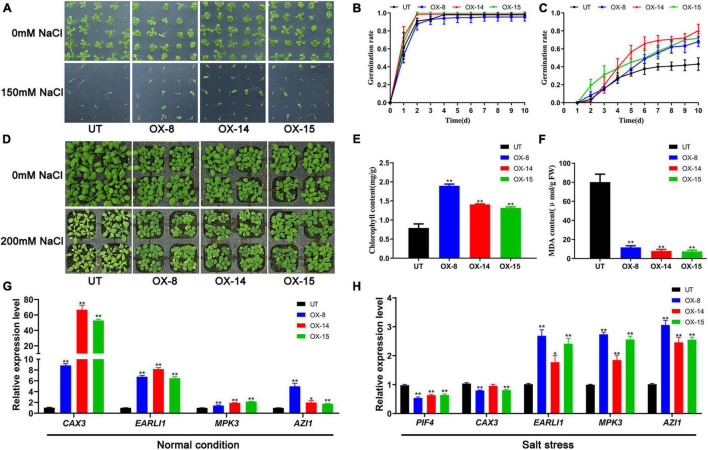
*GmMYB133* confers plant tolerance to salt stress in *Arabidopsis*. **(A)** Photographs of seed germination for UT plant and three transgenic lines overexpressing *GmMYB133* under normal condition and salt stress (150 mM NaCl), which were taken 10 days after culturing under the condition of 16/8 h (light/dark) at 22°C. **(B,C)** Germination assay of UT and three transgenic lines under normal condition **(B)** and salt stress **(C)**. The seeds were sowed and cultured on MS solid medium supplied with or without 150 mM NaCl, and the germination rates were monitored for 10 days. **(D)** Photograph of 30-day-old plants for UT and three transgenic lines under normal condition and salt stress. The 21-day-old plants were exposed to 200 mM NaCl and normal condition for 9 days. **(E,F)** Quantitative analysis of total chlorophylls **(E)** and MDA **(F)** in UT and three transgenic lines under normal condition and salt stress. **(G,H)** The expression patterns of salt tolerance-associated genes in UT and three transgenic *Arabidopsis* lines under normal condition **(G)** and salt stress **(H)**. The genes include *AtCAX3*, *AtEARLI1*, *AtMPK3*, *AtAZI1*, and *AtPIF4*. Total RNAs were exacted from 14-day-old transgenic seedlings and UT. Values were normalized against the gene *AtACTIN8*, and the expression level in UT was set as 1. Error bars in panels **(B,C,E–H)** indicate SE of three biological and technical replicates, and significant differences are denoted by asterisks: *p < 0.05, **p < 0.01.

Since *GmMYB133* showed salt-stress-inducible expression in soybean, its effect on plant tolerance to salt stress was investigated using the transgenic *Arabidopsis* with *GmMYB133* overexpression. First, seeds of three transgenic lines and UT control were sown on solid MS medium supplemented with or without 150 mM NaCl, and then germination rates were dynamically surveyed from 1 to 10 days after vernalization treatment. As shown in Figures 4A,B, a similar germination tendency was observed between transgenic seeds and UT control under normal conditions, and germination rates reached nearly 100% at 4 days after vernalization treatment. However, in the presence of 150 mM NaCl, seed germination was obviously enhanced by *GmMYB133* overexpression. As shown in Figures 4A,C, the germination rates for three transgenic lines were up to 68.0%–80.3%, whereas UT control only showed a 43.0% germination rate. Furthermore, phenotypic and physiological analyses of transgenic plants and UT control were performed in the presence of 200 mM NaCl. Consequently, the *GmMYB133*-overexpressing lines were insensitive to salt stress as compared with UT control. As indicated in Figures 4D,E, the leaves of three transgenic lines were greener than the UT control, and the content of total chlorophylls was significantly increased by *GmMYB133* overexpression with 1.7–2.4-fold changes. Consistently, the MDA contents in the three transgenic lines were decreased to 9.3%–14.6% of UT control (Figure 4F). These results suggested that *GmMYB133* might confer plant tolerance to salt stress in *Arabidopsis*. Additionally, transgenic plants and UT control were subjected to cold stress (−9°C) for 1 h, and phenotypic difference and MDA content were investigated accordingly. As shown in Supplementary Figure 3, no significant difference was observed in the aspects of phenotype and MDA content.

To provide some hints about how *GmMYB133* can respond to salt stress, four positive regulators for salt tolerance were chosen to conduct expression analysis using the qRT-PCR approach, including *AtCAX3*, *AtEARLI1*, *AtMPK3*, and *AtAZI1*. Consequently, the expressions of the four genes were significantly enhanced by *GmMYB133* overexpression to a different extent. Especially, the two genes *AtCAX3* and *AtEARLI1* were transcriptionally elevated by 8.9–66.8- and 6.2–8.2-fold changes, respectively. Previous studies indicated that *AtPIF4* is not only involved in the regulation of light-associated development but also acts as a negative regulator in response to salt stress. Furthermore, the expressions of the above four positive regulators and *AtPIF4* were investigated in the transgenic lines with *GmMYB133* overexpression and UT control under salt stress (150 mM NaCl). Consistently, *AtPIF4* was transcriptionally decreased by *GmMYB133* overexpression under salt stress, while the expressions of *AtEARLI1*, *AtMPK3*, and *AtAZI1* were remarkably increased in the transgenic lines. However, no change or a slight decrease was observed for *AtCAX3* between the transgenic lines and UT control under salt stress. Further observation indicated that the expression of *AtCAX3* was remarkably increased in UT control under salt stress (Figure 4H and Supplementary Figure 4), which is consistent with the previous report that *AtCAX3* expression was strongly induced by salt stress (Shigaki and Hirschi, 2000). These results implied that *GmMYB133* might confer plant tolerance to salt stress through regulating the expressions of salt tolerance-related genes such as *AtEARLI1*, *AtMPK3*, and *AtAZI1* in *Arabidopsis*.

### *GmMYB133* Performs Its Functions Directly Through Regulating the Expression of *AtPRR5* in *Arabidopsis*

**FIGURE 5:**
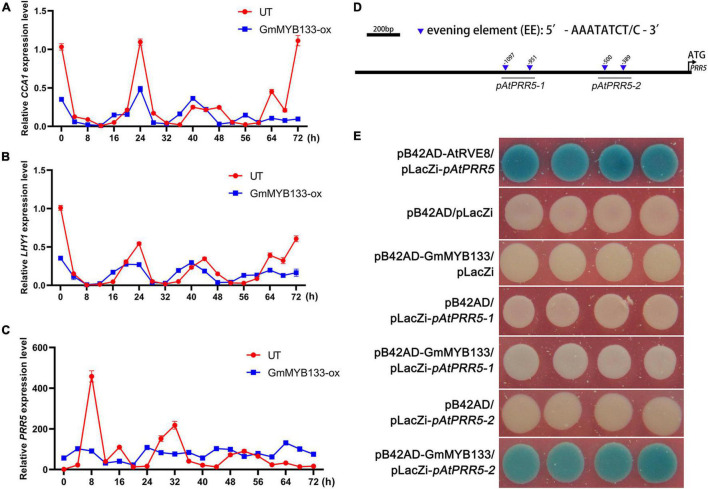
GmMYB133 might affect hypocotyl elongation and salt tolerance directly through regulating the expression of *AtPRR5* in *Arabidopsis*. **(A–C)** Diurnal expression patterns of three core oscillator genes *AtCCA1* **(A)**, *AtLHY1* **(B)**, and *AtPRR5* **(C)** in UT plant and the mix of three transgenic lines overexpressing *GmMYB133* (OX-8, OX-14, and OX-15). Ten-day-old *Arabidopsis* seedlings were transferred into the condition of constant light, and the time course was set every 4 h from 0 h until 72 h. Total RNAs were exacted from transgenic seedlings and UT. Values were normalized against the gene *AtACTIN8*, and the expression level in UT at 0 h was set as 1. Error bars in panel **(A–C)** indicate the SE of three biological and technical replicates. **(D)** The schematic diagram of *GmMYB133* promoter. The downward triangles indicate the evening element AAATATCT/C, and the number shows the start site of each element. Black lines labeled with *pAtPRR5-1* or *pAtPRR5-2* represent the examined regions for the Y1H assay. **(E)** Interaction assay between GmMYB133 and *AtPRR5* promoter (*pAtPRR5-1* and *pAtPRR5-2*) using the Y1H approach. Four plasmid combinations were set as negative controls (pB42AD/pLacZi, pB42AD-GmMYB133/pLacZi, pB42AD/pLacZi-*pAtPRR5-1*, and pB42AD/pLacZi-*pAtPRR5-2*) and one combination (pB42AD-AtRVE8/placZi-*AtPRR5*) as positive control.

It has been known that hypocotyl elongation and salt tolerance are controlled by the circadian clock (Rawat et al., 2011; Nakamichi et al., 2016; Gray et al., 2017). To explore if *GmMYB133* affects the circadian clock, the rhythmic expressions of three core oscillator genes (*AtCCA1*, *AtLHY1*, and *AtPRR5*) were investigated in *GmMYB133*-overexpressing *Arabidopsis* lines and UT control under continuous light using the qRT-PCR approach. Consequently, all the examined oscillator genes sustained approximately 24-h circadian rhythm in UT control. However, overexpression of *GmMYB133* obviously altered the rhythmic expressions of *AtCCA1*, *AtLHY1*, and *AtPRR5*. As shown in Figures 5A,B, *AtCCA1* and *AtLHY1* showed a 4-h rhythmic phase shift and reduced oscillation amplitude in transgenic lines as compared with type. Noticeably, the rhythmic expression of *AtPRR5* was extremely disrupted by *GmMYB133* overexpression. As indicated in Figure 5C, the oscillation amplitude of *AtPRR5* was remarkably reduced by *GmMYB133* overexpression, and a relatively stable expression level was observed for *AtPRR5* in *GmMYB133*-overexpressing *Arabidopsis* lines. On the whole, the trough level of *AtPRR5* was elevated by *GmMYB133* overexpression. These results indicated that *GmMYB133* might serve as a regulator of core circadian oscillators, especially *AtPRR5*, to control diverse biological processes.

Since the expression level and endogenous rhythm of *AtPRR5* were significantly altered by *GmMYB133* overexpression in *Arabidopsis*, it is worth exploring if *GmMYB133* acts as a transcription factor to directly regulate the expression of *AtPRR5*. First, two CCA1-like protein binding sites (AAATATCT and AAATATCC) were observed in the promoter region of the gene *AtPRR5* (Figure 5D). Thus, Y1H assay was performed using *GmMYB133* as bait and two fragments of *AtPRR5* promoter (named as *pAtPRR5-1* and *pAtPRR5-2*) as preys. Consequently, like the positive control (pB42AD-AtRVE8/placZi-*pAtPRR5*), blue colonies were shown when *GmMYB133* served as bait and the two fragments of *AtPRR5* promoter (*pAtPRR5-1* and *pAtPRR5-2*) as preys. As shown in Figure 5E, the colonies harboring the plasmid combination of GmMYB133 and *pAtPRR5-2* showed strong blue color, while a very weak blue color was observed for the colonies with the plasmid combination of *GmMYB133* and *pAtPRR5-1*. No blue color appeared in the four negative controls (pB42AD/pLacZi, pB42AD-GmMYB133/pLacZi, pB42AD/pLacZi-*pAtPRR5-1*, and pB42AD/pLacZi-*pAtPRR5-2*) (Figure 5E). These results indicated that physical interaction could occur between GmMYB133 protein and *AtPRR5* promoter.

GmMYB133 can affect the expressions of *AtPIF4*, *AtBBX24*, *AtIAA19*, *AtIAA29*, *AtSAUR21*, *AtSAUR26*, *AtCAX3*, *AtEARLI1*, *AtMPK3*, and *AtAZI1*. To explore whether GmMYB133 acts as a transcription factor to directly target these genes, we first predicted the binding site of CCA1-like MYBs, the evening element, in their promoter regions using the online program PlantPAN 3.0. As shown in Supplementary Figure 5A, all the ten genes harbored 1–4 evening element(s) in their promoter regions. Furthermore, the Y1H assay was performed using GmMYB133 as bait and the promoter fragments with evening elements as prey. Consequently, no blue colony was observed when GmMYB133 served as bait and the promoter fragments of the above ten genes as prey, while blue colonies appeared for the positive control (Supplementary Figure 5B).

**FIGURE 6:**
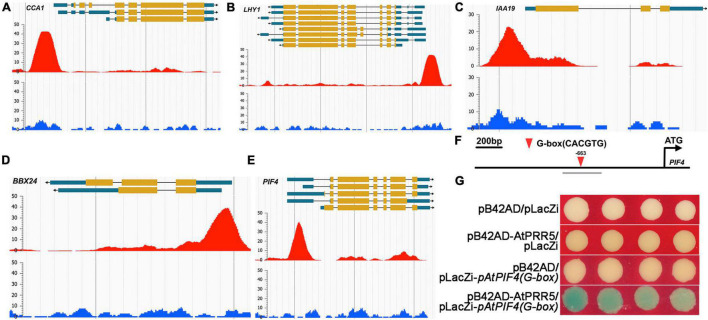
AtPRR5 might directly target circadian, light-responsive, and auxin-associated genes. **(A–E)** Enrichment peaks for the binding of AtPRR5 to the genomic regions of the circadian clock, light-responsive, and auxin-associated genes. The ChIP-Seq data of AtPRR5 were retrieved against *Arabidopsis thaliana* using AtPRR5 as assayed protein through the Encyclopedia of Plant Genome (ENPG) database (www.plantseq.org). Enrichment peaks for the binding of AtPRR5 to the genomic regions of *AtCCA1*, *AtLHY1*, *AtPIF4*, *AtIAA19*, and *AtBBX24* were visualized through the program ENPG using the default parameters. The red peaks represent significant occupies of AtPRR5 at its target genes, and blue signals indicate background derived from input control. Gene structures are shown on the top of each panel. **(F)** The schematic diagram of the *AtPIF4* promoter. The downward triangle indicates the G-box (CACGTG), and the number shows the start site of the G-box element. The gray line labeled with *pAtPIF4(G-box)* represents the examined region for the Y1H assay. **(G)** Interaction assay between AtPRR5 and *AtPIF4* promoter using Y1H approach. Three plasmid combinations were set as negative controls [pB42AD/pLacZi, pB42AD-AtPRR5/pLacZi, and pB42AD/pLacZi-*pAtPIF4(G-box)*].

To further address *GmMYB133*-mediated mechanisms that control hypocotyl elongation and response to salt stress in *Arabidopsis*, we retrieved the ChIP-Seq data of *AtPRR5* (Nakamichi et al., 2012) to obtain its target clients through the online program ENPG (see text footnote 6). Consequently, the peaks of the AtPRR5 binding site were observed for two rhythm-associated genes (*AtCCA1* and *AtLHY1*), two light-responsive genes (*AtPIF4* and *AtBBX24*), and *AtIAA19* (Figures 6A–E). Further observation indicated that the peaks were mainly distributed in the promoter and/or UTR regions. Furthermore, *AtPIF4* was chosen as a representative to confirm the binding of AtPRR5 using Y1H assay. AtPRR5 usually prefers to bind to the element G-box (CACGTG), and it was observed that *AtPIF4* harbored one G-box element in its promoter region (Figure 6F). Y1H assay indicated that blue colonies appeared when AtPRR5 served as bait and the fragment of *AtPIF4* promoter as prey, while no blue colony was observed for the negative control (Figure 6G). These results suggested that *GmMYB133* might affect hypocotyl elongation and salt tolerance directly through regulating the expression of *AtPRR5*, which subsequently alters the transcriptional accumulations of its direct targets such as *AtPIF4*, *AtBBX24*, and *AtIAA19*.

## Discussion

*RVE* genes are generally recognized as core circadian components to activate the expression of their downstream oscillator genes, therefore enabling plants to properly respond to the day/night time or adverse environmental stimuli (Gray et al., 2017; Chen S. et al., 2020; Li X. et al., 2020; Badhan et al., 2021). *RVE* genes have been documented to participate in the regulation of the first wave of heat shock-induced gene expression (Li et al., 2019a), cold tolerance (Chen S. et al., 2020), anthocyanin accumulation (Li X. et al., 2020), hypocotyl elongation (Rawat et al., 2011), growth of juvenile, and adult plants (Gray et al., 2017). However, the functional roles of *RVE* genes remained unclear in soybean. Previously, we reported that *GmMYB133* affects isoflavonoid biosynthesis in soybean (Bian et al., 2018). In this study, we provided studies to support that *GmMYB133* might act as an *RVE* transcription factor to regulate hypocotyl elongation and salt tolerance, which will enhance our understanding about the regulatory roles of *RVE* genes in soybean.

Sequence analysis showed that *GmMYB133* is homologous to the *RVE8* clade genes of *Arabidopsis* such as *AtRVE3*, *AtRVE4*, *AtRVE5*, *AtRVE6*, and *AtRVE8* (Figure 1). In this study, we provided three aspects of studies to support that *GmMYB133* acts as an *RVE8* clade gene to negatively regulate plant growth. First, overexpression of *GmMYB133* in *Arabidopsis* altered multiple circadian clock outputs such as decreased hypocotyl lengths of seedlings, short-petiole leaf, and late flowering (Figure 3). These observations are in agreement with the previous reports that *AtRVE8*-overexpressing *Arabidopsis* plants showed a late flowering phenotype (Rawat et al., 2011), while simultaneous mutation of *RVE4*, *RVE6*, and *RVE8* in *Arabidopsis* facilitated the growth of hypocotyl and leaf petiole (Gray et al., 2017). It is not surprising since the *RVE8* clade genes inhibit growth rate and adult cell size. Second, hypocotyl growth is regulated by a series of internal and external cues such as hormone and light (Reed et al., 2018; Kelly et al., 2021). In this study, five auxin-associated genes and two light-responsive genes *AtPIF4* and *AtBBX24* were, in fact, repressed by *GmMYB133* overexpression in *Arabidopsis* (Figure 3F). These results are consistent with the previous reports that *AtPIF4* was largely responsible for the effects of *AtRVE4*, *AtRVE6*, and *AtRVE8* on hypocotyl growth (Gray et al., 2017), while mutation of *AtBBX24* in *Arabidopsis* led to shorter hypocotyl (Jiang et al., 2012). Third, overexpression of *GmMYB133* not only altered the expression intensity of three central oscillators such as *AtCCA1*, *AtLHY1*, and *AtPRR5* in *Arabidopsis* but also caused a rhythmic phase shift. Thus, we assumed that *GmMYB133* might regulate the photoperiodic plant growth (hypocotyl elongation, leaf petioles, and flowering) by affecting the expressions of the central oscillators such as *AtCCA1*, *AtLHY1*, and *AtPRR5*. Previous studies indicated that *AtRVE4*, *AtRVE6*, and *AtRVE8* negatively affect hypocotyl elongation, while *AtRVE3* and *AtRVE5* do not contribute significantly to the effect (Gray et al., 2017). Also, *AtRVE8* promotes the expression of *AtPRR5* in *Arabidopsis* (Rawat et al., 2011). Although *GmMYB133* showed maximum identity with *AtRVE5* (56.86%) at the protein level in *Arabidopsis* (Figure 1), our results supported that *GmMYB133* might perform functions similar to *AtRVE4*, *AtRVE6*, or *AtRVE8*, at least in the aspect of hypocotyl elongation. Noticeably, the rhythmic expressions of *AtCCA1*, *AtLHY1*, and *AtPRR5* were perturbated by the overexpression of *GmMYB133*. However, no typical rhythmic expression was observed for *GmMYB133* itself (Figure 2I). Previous report indicated that *AtRVE3*, *AtRVE4*, and *AtRVE8*, but not *AtRVE5* and *AtRVE6*, showed rhythmic expression in *Arabidopsis* seedlings, and *AtRVE4*, *AtRVE6*, and *AtRVE8* might perform partially redundant functions in speeding up the internal clock pace (Rawat et al., 2011). We speculated that *GmMYB133* possibly works together with clock-associated factor (s) to control clock pace.

Recently, several studies indicated that *RVE* genes play important roles in plant response to abiotic stress including heat, cold, and drought. For example, *A*t*RVE8* and its close homolog *AtRVE4* enabled to affect plant thermotolerance by regulating the first wave of heat shock-induced gene expression in *Arabidopsis* (Li et al., 2019a); overexpression of *SgRVE6* in tobacco conferred cold tolerance (Chen S. et al., 2020); drought tolerance of CRISPR-edited *RVE7* protoplast was decreased in chickpea (Badhan et al., 2021). To our best knowledge, however, it remains unknown if *RVE* genes are involved in the regulation of salt stress tolerance. In this study, *GmMYB133* showed a salt stress-inducible expression pattern (Figure 2K). Meanwhile, overexpression of *GmMYB133* in *Arabidopsis* enhanced seed germination in salt stress (Figures 4A–C), and phenotypic and physiological analysis indicated that *GmMYB133* could improve plant tolerance to salt stress in *Arabidopsis* (Figures 4D,E). Moreover, four salt tolerance-associated genes (*AtCAX3*, *AtEARLI1*, *AtAZI1*, and *AtMPK3*) were transcriptionally elevated by overexpression of *GmMYB133* (Figure 4F). It was reported that *AtCAX3* expression was strongly induced by salt stress, and *cax3* mutant showed more sensitivity to salt stress (Zhao et al., 2008); *EARLI1* in *Arabidopsis* greatly facilitated seed germination and seedling development in salt stress (Xu et al., 2011); *AtAZI1* overexpression in *Arabidopsis* strongly conferred plant tolerance to high-salinity stress, and *AtMPK3* acted as a positive regulator of *AtAZI1* expression (Pitzschke et al., 2014). Thus, we speculated that *GmMYB133* might be involved in the regulation of plant tolerance to salt stress through regulating the salt tolerance-associated genes in *Arabidopsis*. Noticeably, *GmMYB133* can simultaneously coordinate the activation of the salt-tolerance genes and the inactivation of the light-responsive and auxin-associated genes (Figures 3F, 4G). It is reasonable since MYBs usually form a complex with themselves or other players to co-regulate diverse developmental processes or respond to various stresses.

*PRR5* serves as the core oscillator gene to be involved in the regulation of a wide range of biological processes, including ABA biosynthesis and signaling, root cell proliferation, inhibitory expression of morning-phased clock genes, flowering, photoperiod-responsive growth, and abiotic stress tolerance such as cold, drought, and salt stresses (Nakamichi et al., 2007, 2012, 2016; Rawat et al., 2011; Li et al., 2019b; Yang et al., 2021). In this study, the clock rhythm of *AtPRR5* was extremely perturbed by *GmMYB133* overexpression, and the trough level of *AtPRR5* was elevated by *GmMYB133* overexpression (Figure 5C). Moreover, the Y1H assay indicated that GmMYB133 can bind to the promoter region of *AtPRR5* (Figures 5D,E). These observations are in agreement with the previous study that AtRVE8 enabled to directly target *AtPRR5* and disturbed its rhythmic expression (Rawat et al., 2011). Our results suggested that *GmMYB133* and *PRR5* possibly constitute a regulatory module to regulate plant growth and stress tolerance. Increasing evidence indicated that *PIF4* serves as a molecular hub to positively affect hypocotyl elongation, flowering, and petiole growth by integrating the effects of light and hormones (Kunihiro et al., 2011; Galvao et al., 2019; Rosado et al., 2019; Li et al., 2021). Also, it was observed that the leaves of *Arabidopsis pif4-1* mutant showed lower ion leakage under salt stress, whereas overexpression of *AtPIF4* caused young seedlings more sensitive to salt stress (Sakuraba et al., 2017). In this study, *GmMYB133* overexpression led to several phenotypes opposing to the ones of *AtPIF4* mutation such as decreased hypocotyl length of seedlings, short-petiole leaf, and late flowering as well as improvement of plant tolerance to salt stress (Figures 3, 4), and, indeed, *AtPIF4* were transcriptionally decreased in *GmMYB133*-overexpressing *Arabidopsis* under normal condition and salt stress (Figures 3F, 4H). Intriguingly, the retrieved ChIP-Seq data (Nakamichi et al., 2012) and our Y1H assay showed that *AtPIF4* and *AtIAA19* can be directly targeted by AtPRR5 (Figures 6A–E). Moreover, it was reported that *AtPRR5* serves as transcriptional repressors of *AtPIF4* to regulate photoperiodic growth (Takase et al., 2013; Zhu et al., 2016). Thus, we assumed that the regulatory module GmMYB133-*AtPRR5* and the integrator *AtPIF4* might constitute a triple hierarchy architecture to govern the expressions of their downstream genes, therefore, affecting hypocotyl elongation, leaf petiole growth, flowering, and salt stress tolerance in *Arabidopsis*.

## Conclusion

*GmMYB133* is homologous to the *RVE8* clade genes of *Arabidopsis*. Like the *RVE* genes in *Arabidopsis*, *GmMYB133* affected hypocotyl elongation, leaf petiole growth, and flowering in *Arabidopsis*. More importantly, *GmMYB133* conferred plant tolerance to salt stress. Furthermore, a regulatory module *GmMYB133-PRR5-PIF4* was proposed to regulate hypocotyl elongation, leaf petiole growth, flowering, and salt stress tolerance in *Arabidopsis*. These findings laid a foundation to further address the functional roles of *GmMYB133* and its regulatory mechanisms in soybean.

## Data Availability Statement

The datasets presented in this study can be found in online repositories. The names of the repository/repositories and accession number(s) can be found in the article/Supplementary Material.

## Author Contributions

XL and SB designed the experiments. BS, WW, and SX performed the experiments. JC and RL performed the data analysis. XL, SB, and JC wrote the manuscript. All authors read and approved the final manuscript.

## Conflict of Interest

The authors declare that the research was conducted in the absence of any commercial or financial relationships that could be construed as a potential conflict of interest.

## Publisher’s Note

All claims expressed in this article are solely those of the authors and do not necessarily represent those of their affiliated organizations, or those of the publisher, the editors and the reviewers. Any product that may be evaluated in this article, or claim that may be made by its manufacturer, is not guaranteed or endorsed by the publisher.

## Footnotes

1
https://phytozome-next.jgi.doe.gov

2
https://www.arabidopsis.org

3
https://www.ebi.ac.uk/Tools/msa/muscle/

4
https://www.graphpad.com/

5
http://plantpan.itps.ncku.edu.tw/index.html

6
www.plantseq.org

## Supplementary Material

Supplementary Figure 1 Sequence analysis of GmMYB133 and its homologous proteins in soybean and *Arabidopsis*. **(A)** Sequence alignment of GmMYB133 protein with its homologs in other plant species. Amino-acid sequences were aligned by MEGA X and then imported into DNAMAN for shading. Protein homology ≥ 33% is shown as yellow, ≥ 50% as blue, ≥ 75% as pink, and 100% as black. The conserved MYB domain is marked with the black line, while the active site of the consensus sequence SHAQ(Y/F)F is labeled with the star. **(B)** Protein homology analysis of GmMYB133 with its homologs.

Supplementary Figure 2 Histochemical analysis and GUS activity assay. **(A)** Histochemical staining and **(B)** fluorometric GUS assay of transgenic *Arabidopsis* with *pGmMYB133:GUS* under salt and drought stress. Seven-day-old transgenic *Arabidopsis* seedlings with *pGmMYB133:GUS* were subjected to salt (150 mM NaCl) or drought (300 mM mannitol) stress for 3 days. Scale bars indicate 1 mm. **(C)** The expression of *GUS* gene in young leaves of above transgenic *Arabidopsis* seedlings under salt and drought stress. Values were normalized against the gene *AtACTIN8*, and the expression level under the normal condition was set as 1. Error bars indicate SE of three biological and technical replicates, and significant differences are denoted by asterisks: ***p* < 0.01.

Supplementary Figure 3 *GmMYB133* might not confer plant tolerance to cold stress. **(A)** Photographs of 14-day-old seedlings of untransformed (UT) control and three transgenic lines overexpressing *GmMYB133* under normal condition (left panel) and cold stress (right panel). Scale bars indicate 2 cm. **(B)** MDA contents of UT and transgenic lines under cold stress. Error bars in **(B)** indicate SE of three biological and technical replicates, and significant differences are denoted by asterisks: ***p* < 0.01.

Supplementary Figure 4 Comparison of *AtCAX3* expression under normal condition and salt stress. Statistical significance of the data was analyzed using one-way ANOVA with Duncan. Error bars refer to SE, and lowercase letters above all the bars indicate statistically significant differences.

Supplementary Figure 5 GmMYB133 fails to bind to the promoters of light-responsive, auxin-related, and salt tolerance-associated genes. **(A)** The schematic diagrams of the promoters of the light-responsive, auxin-associated, and salt tolerance-related genes such as *AtPIF4*, *AtBBX24*, *AtIAA19*, *AtIAA29*, *AtSAUR21*, *AtSAUR26, AtCAX3*, *AtEARLI1*, *AtMPK3*, and *AtAZI1*. The downward triangles indicate the evening elements (EE), and the number shows the start site of each element. Gray lines represent the examined regions for Y1H assay. **(B)** Interaction assay between GmMYB133 and the promoters of the light-responsive, auxin-associated, and salt tolerance-related genes using the Y1H approach. Twelve plasmid combinations were set as negative controls [pB42AD/pLacZi, pB42AD-GmMYB133/pLacZi, pB42AD/pLacZi-*pAtPIF4(EE)*, pB42AD/pLacZi-*pAtBBX24*, pB42AD/pLacZi- *pAtIAA19*, pB42AD/pLacZi-*pAtIAA29*, pB42AD/pLacZi-*pAtSAUR21*, pB42AD/pLacZi-*pAtSAUR26*, pB42AD/pLacZi-*pAtCAX3*, pB42AD/pLacZi-*pAtEARLI1*, pB42AD/pLacZi-*pAtMPK3*, and pB42AD/pLacZi-*pAtAZI1*] and one positive control (pB42AD-AtRVE8/placZi-*pAtPRR5*).
