# Supplementary figures and images for: Soybean GmMYB133 Inhibits Hypocotyl Elongation and Confers Salt Tolerance in Arabidopsis
Source: Front Plant Sci. 2021 Dec 23;12:764074. doi: 10.3389/fpls.2021.764074 (PMC8732865; doi:10.3389/fpls.2021.764074)

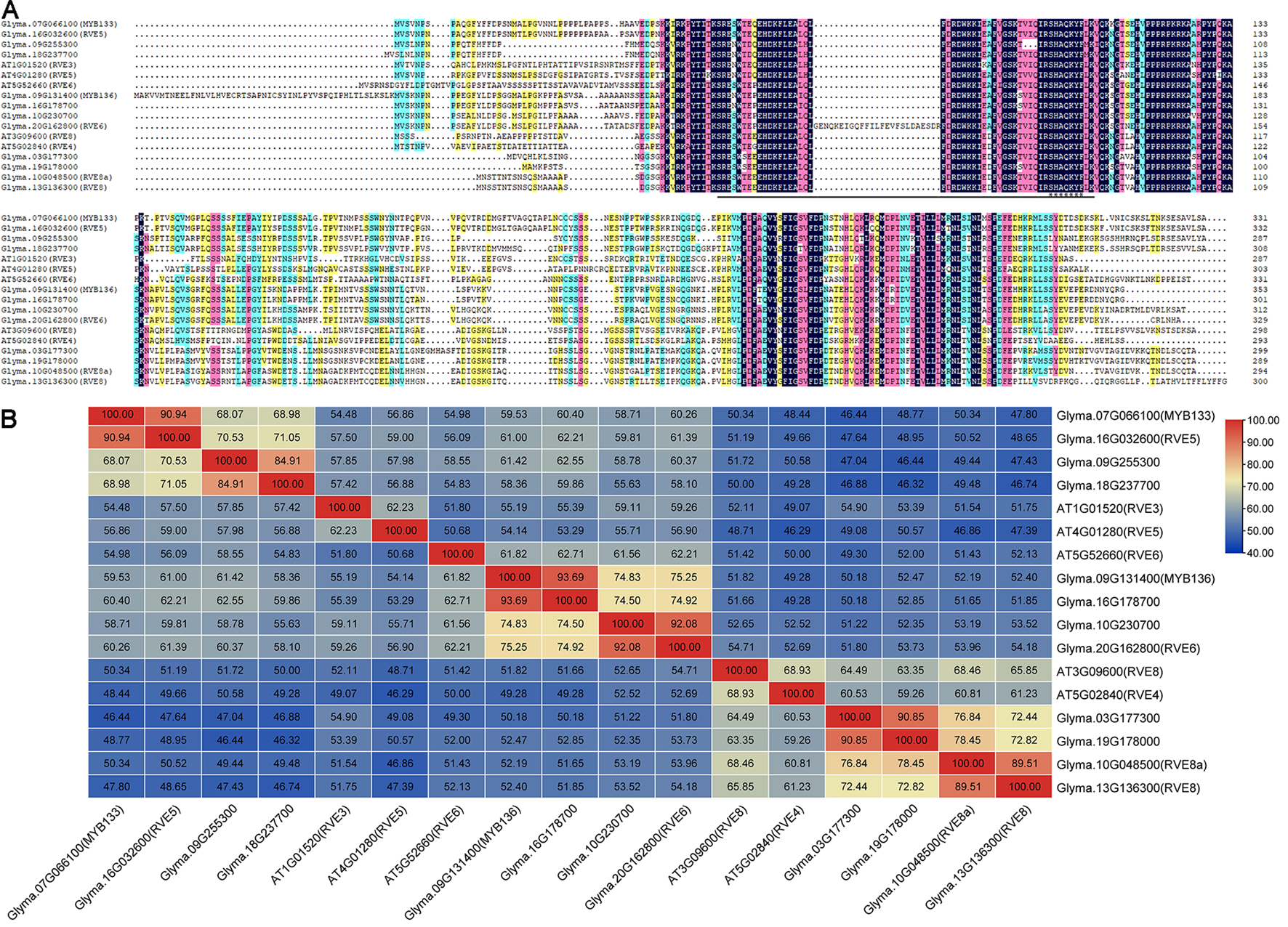

Supplement: Supplementary Figure 1 — Sequence analysis of GmMYB133 and its homologous proteins in soybean and Arabidopsis. (A) Sequence alignment of GmMYB133 protein with its homologs in other plant species. Amino-acid sequences were aligned by MEGA X and then imported into DNAMAN for shading. Protein homology ≥ 33% is shown as yellow, ≥ 50% as blue, ≥ 75% as pink, and 100% as black. The conserved MYB domain is marked with the black line, while the active site of the consensus sequence SHAQ(Y/F)F is labeled with the star. (B) Protein homology analysis of GmMYB133 with its homologs. [file Image_1.TIF]

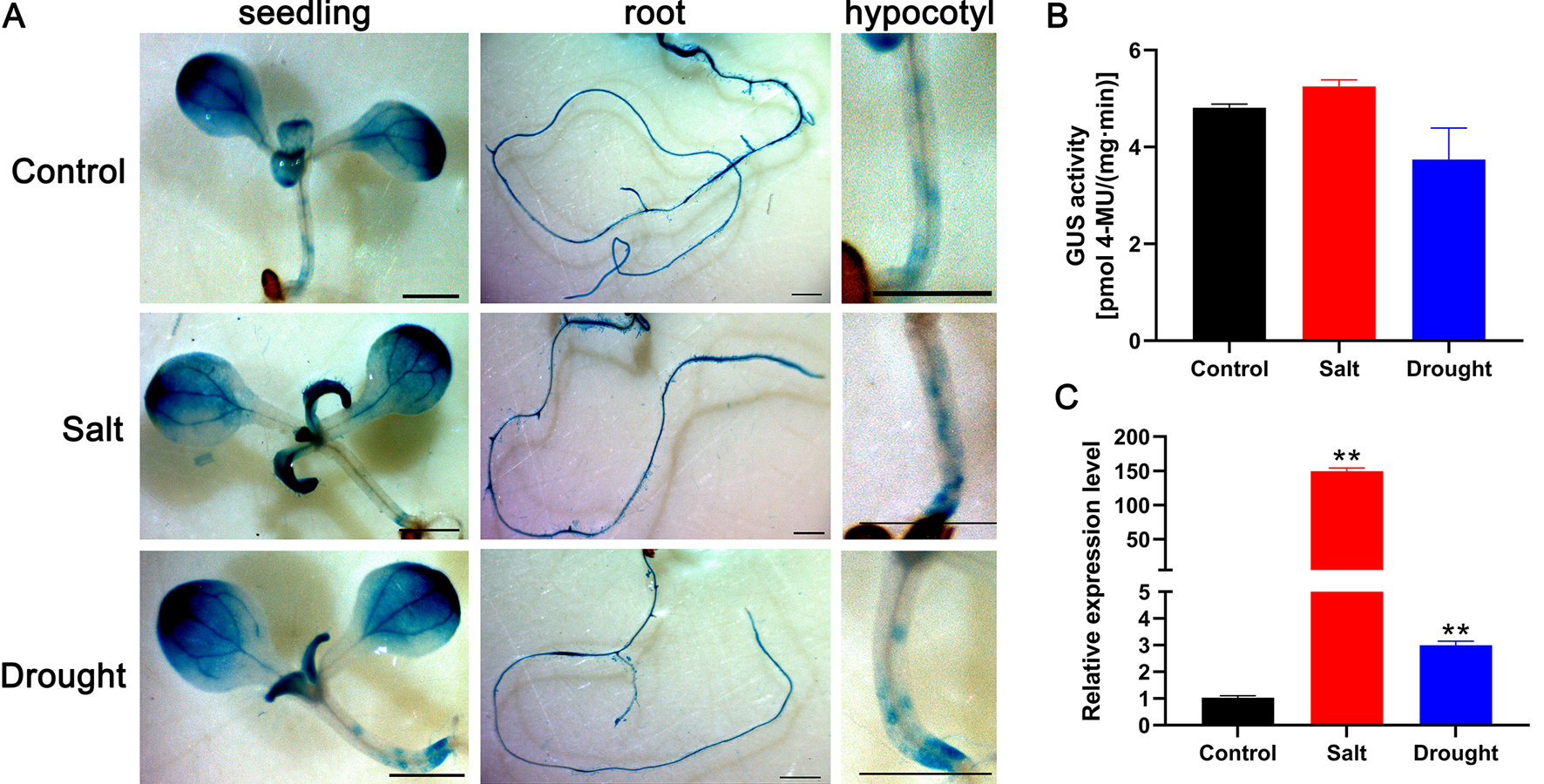

Supplement: Supplementary Figure 2 — Histochemical analysis and GUS activity assay. (A) Histochemical staining and (B) fluorometric GUS assay of transgenic Arabidopsis with pGmMYB133:GUS under salt and drought stress. Seven-day-old transgenic Arabidopsis seedlings with pGmMYB133:GUS were subjected to salt (150 mM NaCl) or drought (300 mM mannitol) stress for 3 days. Scale bars indicate 1 mm. (C) The expression of GUS gene in young leaves of above transgenic Arabidopsis seedlings under salt and drought stress. Values were normalized against the gene AtACTIN8, and the expression level under the normal condition was set as 1. Error bars indicate SE of three biological and technical replicates, and significant differences are denoted by asterisks: **p < 0.01. [file Image_2.TIF]

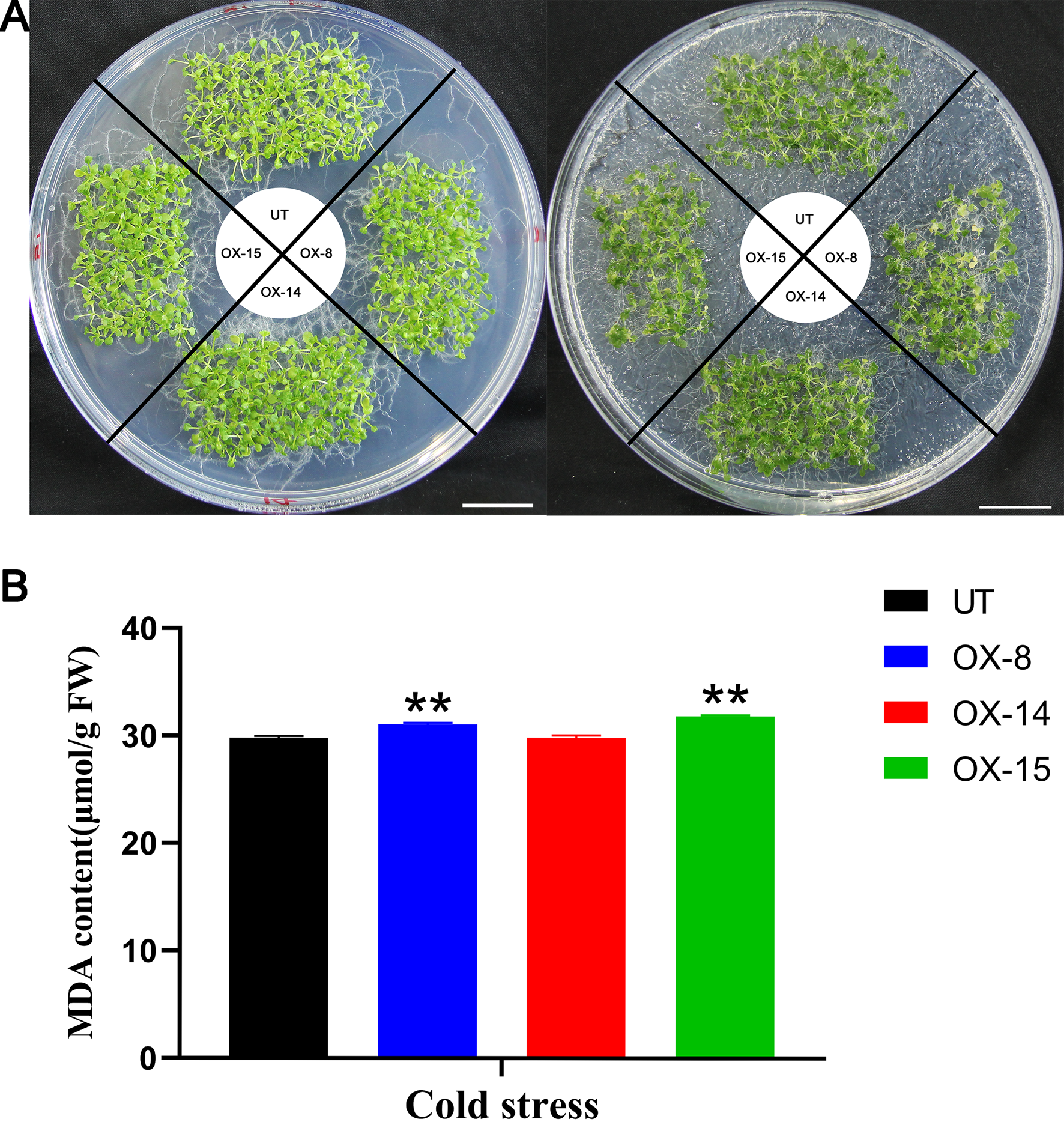

Supplement: Supplementary Figure 3 — GmMYB133 might not confer plant tolerance to cold stress. (A) Photographs of 14-day-old seedlings of untransformed (UT) control and three transgenic lines overexpressing GmMYB133 under normal condition (left panel) and cold stress (right panel). Scale bars indicate 2 cm. (B) MDA contents of UT and transgenic lines under cold stress. Error bars in (B) indicate SE of three biological and technical replicates, and significant differences are denoted by asterisks: **p < 0.01. [file Image_3.TIF]

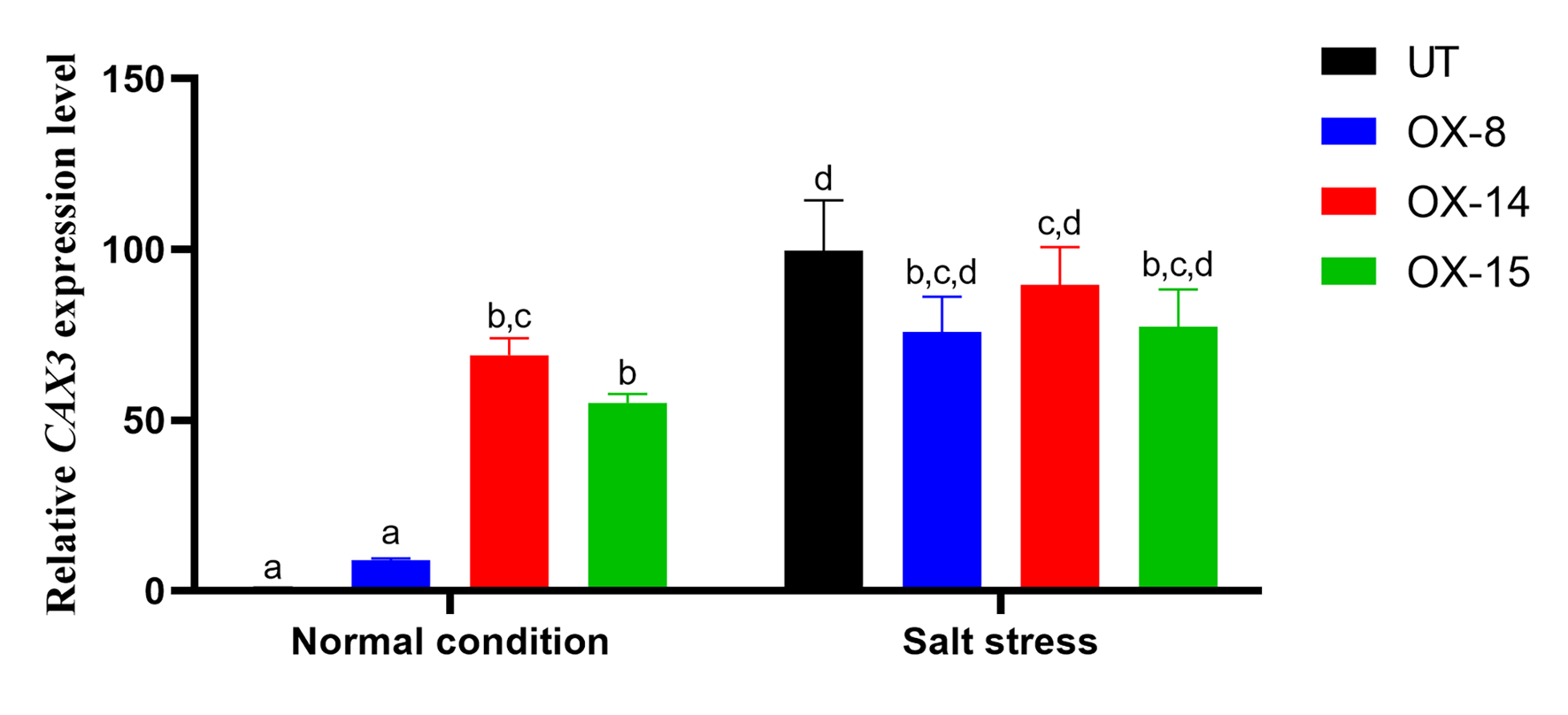

Supplement: Supplementary Figure 4 — Comparison of AtCAX3 expression under normal condition and salt stress. Statistical significance of the data was analyzed using one-way ANOVA with Duncan. Error bars refer to SE, and lowercase letters above all the bars indicate statistically significant differences. [file Image_4.TIF]

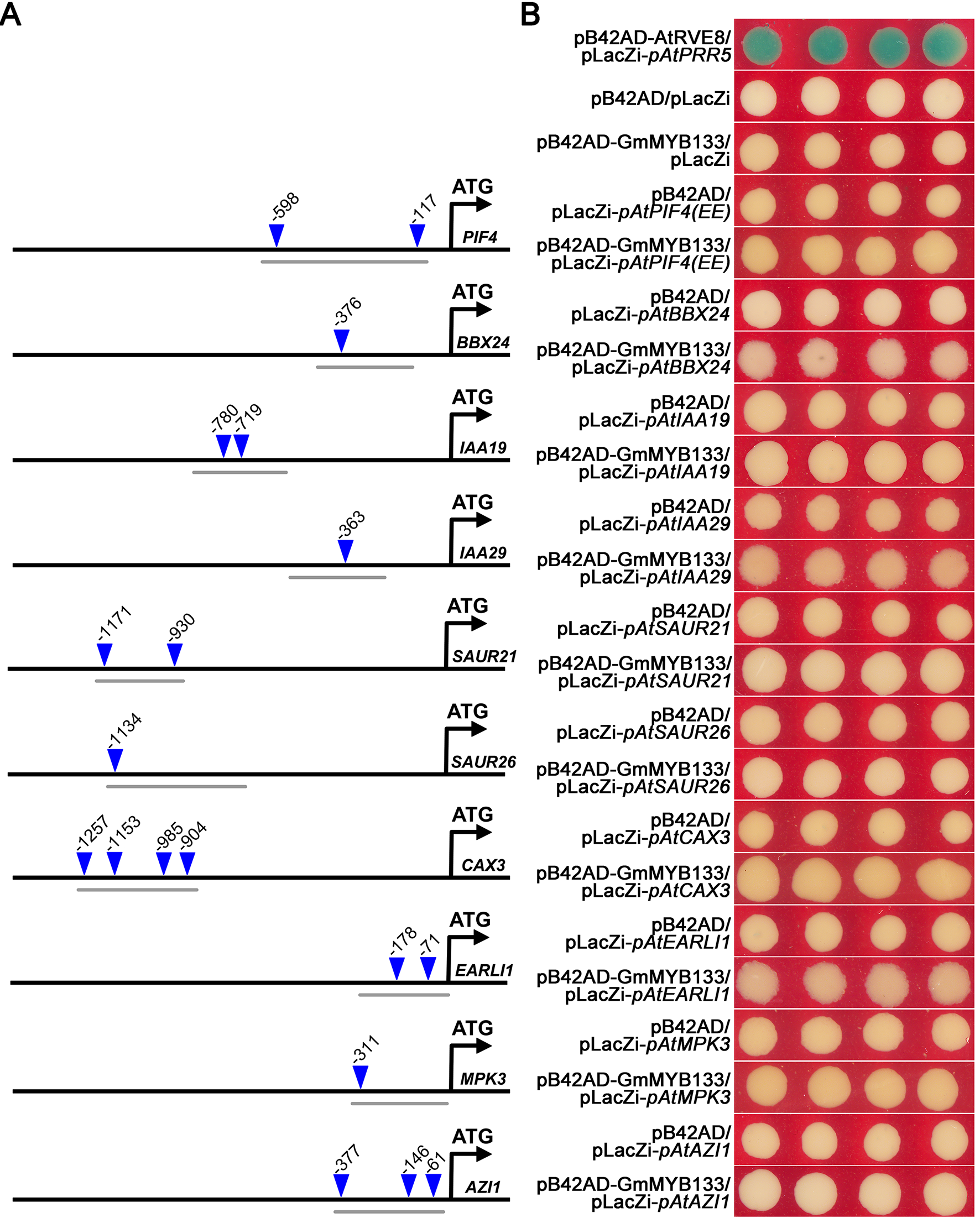

Supplement: Supplementary Figure 5 — GmMYB133 fails to bind to the promoters of light-responsive, auxin-related, and salt tolerance-associated genes. (A) The schematic diagrams of the promoters of the light-responsive, auxin-associated, and salt tolerance-related genes such as AtPIF4, AtBBX24, AtIAA19, AtIAA29, AtSAUR21, AtSAUR26, AtCAX3, AtEARLI1, AtMPK3, and AtAZI1. The downward triangles indicate the evening elements (EE), and the number shows the start site of each element. Gray lines represent the examined regions for Y1H assay. (B) Interaction assay between GmMYB133 and the promoters of the light-responsive, auxin-associated, and salt tolerance-related genes using the Y1H approach. Twelve plasmid combinations were set as negative controls [pB42AD/pLacZi, pB42AD-GmMYB133/pLacZi, pB42AD/pLacZi-pAtPIF4(EE), pB42AD/pLacZi-pAtBBX24, pB42AD/pLacZi- pAtIAA19, pB42AD/pLacZi-pAtIAA29, pB42AD/pLacZi-pAtSAUR21, pB42AD/pLacZi-pAtSAUR26, pB42AD/pLacZi-pAtCAX3, pB42AD/pLacZi-pAtEARLI1, pB42AD/pLacZi-pAtMPK3, and pB42AD/pLacZi-pAtAZI1] and one positive control (pB42AD-AtRVE8/placZi-pAtPRR5). [file Image_5.TIF]
